# Del17p does not always significantly influence the survival of B-cell chronic lymphoproliferative disorders

**DOI:** 10.18632/oncotarget.23261

**Published:** 2017-12-15

**Authors:** Shuhua Yi, Zengjun Li, Dehui Zou, Wenjie Xiong, Heng Li, Rui Cui, Chengwen Li, Yuting Yan, Wei Liu, Rui Lv, Zhen Yu, Weiwei Chen, Yan Xu, Gang An, Huijun Wang, Kun Ru, Tao Cheng, Jianxiang Wang, Lugui Qiu

**Affiliations:** ^1^ State Key Laboratory of Experimental Hematology, Institute of Hematology and Blood Disease Hospital, Chinese Academy of Medical Sciences and Peking Union Medical College, Tianjin, P.R.China; ^2^ Department of Hematology, Tianjin First Center Hospital, Tianjin, P.R.China

**Keywords:** B-cell chronic lymphoproliferative disorders, chronic lymphocytic leukemia, cytogenetic aberration, del 17p, prognosis

## Abstract

B-cell chronic lymphoproliferative disorders (B-CLPD) comprise several entities with indolent clinical manifestations but heterogeneous survival. Cytogenetic aberrations are now the standard prognostic predictors in chronic lymphocytic leukemia (CLL) but have been less investigated in other subtypes of B-CLPD. In this study, we detected cytogenetic aberrations by fluorescence *in situ* hybridization (FISH) in 875 B-CLPD patients, based on a panel probes locating at 13q14, 11q22, 17p13 and CEP12. We identified del17p acted as the independent adverse cytogenetic predictor for overall survival (OS) in CLL. Del13q, del11q and del17p were adverse factors for OS in Waldenström's macroglobulinemia in the univariate analysis but lost their role in the multivariate analysis. Trisomy 12 acted as an independent poor factor for both marginal zone lymphoma (MZL) and unclassified B-CLPD (BCLPD-U) subtype. Del17p did not impact survival in MZL and BCLPD-U patients. These contrasting results indicate different roles of the same cytogenetic aberrations in the pathogenesis of each B-CLPD subtype. As del17p contributed to the poorest survival in CLL and desired extraordinary treatment strategy, the imitation of CLL strategy to other B-CLPD with del17p should be carefully advocated based on this study.

## INTRODUCTION

B-cell chronic lymphoproliferative disorders (B-CLPD) are a group of malignant diseases that are characterized by the accumulation of mature B lymphocyte in the bone marrow, peripheral blood, and lymphoid tissues. B-CLPD comprise several entities with similar clinical manifestations, including chronic lymphocytic leukemia (CLL), which accounts for the majority of disorders, and disorders such as follicular lymphoma (FL), hairy cell leukemia (HCL), splenic marginal zone lymphoma (SMZL), nodal marginal zone lymphoma (NMZL), gastric mucosa-associated lymphoid tissue (MALT) lymphoma, lymphoplasmacytic lymphoma/Waldenström’s macroglobulinemia (LPL/WM) [1], and certain unclassified B-CLPD [2]. B-CLPD has a generally indolent clinical course, but heterogeneity exists in these diseases. For example, a hallmark of CLL is its tremendously variable clinical course, with survival ranging from months to decades. Cytogenetic aberrations detected by fluorescence *in situ* hybridization (FISH) are now the main prognostic predictors in CLL. Approximately 80% of CLL cases harbor recurrent genetic aberrations that can be visualized by FISH using a panel of probes; these aberrations include del13q (RB1/D13S25), del11q (ATM), del17p (TP53), trisomy 12 and IGH translocation (t [14q32]). Deletion of TP53 or ATM is considered an adverse predictor, the sole deletion of 13q14 is considered a favorable predictor, and trisomy 12 is considered intermediate predictors [3–5]. The results of this panel of probes have become important in developing clinical therapeutic strategies.

Because the incidence of other B-CLPDs is relatively lower than that of CLL, the prognostic role of the cytogenetic aberrations in these diseases has not been well established. In particular, no study has systematically investigated the incidence and prognostic value of the CLL FISH panel in patients with other B-CLPD. In the present study, we thus provide this information for a cohort of 875 patients.

## RESULTS

### The clinical characteristics of each B-CLPD subtype

In total, 875 patients were enrolled in this analysis. The distribution of the diagnoses in this cohort was as follows: 458 patients with CLL, 6 with B-cell prolymphocytic leukemia (B-PLL), 24 with HCL, 24 with MZL with bone marrow/peripheral blood involvement, 87 with SMZL, 60 with FL with bone marrow/peripheral blood involvement, 98 with WM, and 118 with unclassified B-CLPD (BCLPD-U). As B-PLL is always presented as aggressive course, we separated it in the following analysis. Among the patients with BCLPD-U, 25 had done the MYD88 L265P mutation test and 4 patients had BRAF V600E, all of which were negative.

The clinical characteristics are summarized in Table 1. All the patients had bone marrow involvement and classified into Stage IV. The median age and the percentage of patients with an age >60 years were higher in the WM and BCLPD-U group. Patients with non-CLL had a higher percentage of B symptoms and a lower median WBC level at diagnosis. The male/female ratio was around (2–4):1, except MZL and FL with near half to half ratio. The frequency of elevated LDH ranged from 11%–29%. In CLL, 69 patients (15.1%) were classified as having low Rai risk status (Rai stage 0); 239 patients (52.3%) with intermediate Rai risk status (Rai stage 1–2); and 149 patients (32.6%) with high Rai risk status (Rai stage 3–4). For the 52 FL patients with FLIPI score available, 13 patients (25.0%) were classified as the low-risk group, 31 patients (59.6%) with intermediate-risk group, while 8 patients (15.4%) in the high-risk group.

**Table 1:** The clinical characteristics of B-CLPD

Clinical characteristics	CLL *N* = 458 (%)	WM *N* = 98 (%)	MZL *N* = 111 (%)	HCL *N* = 24 (%)	FL *N* = 60 (%)	BCLPD-U *N* = 118 (%)
**Median age (range)**	58.0 (26.0–86.0)	61.0 (32.0–87.0)	56.0 (28.0–80.0)	51.0 (33.0–78.0)	45.0 (19.0–85.0)	60.0 (24.0–89.0)
**Age > 60 years**						
**YES**	192 (42.0)	51 (52.0)	36 (32.7)	3 (12.5)	10 (16.7)	74 (45.1)
**NO**	265 (58.0)	47 (48.0)	74 (67.3)	21 (87.5)	50 (83.3)	90 (54.9)
**Sex**						
**Male**	306 (67.0)	72 (73.5)	59 (53.2)	19 (79.2)	30 (50.0)	105 (64.0)
**Female**	151 (33.0)	26 (26.5)	52 (46.8)	5 (20.8)	30 (50.0)	59 (36.0)
**B symptom**						
**YES**	101 (27.1)	28 (32.6)	53 (48.6)	8 (34.8)	21 (37.5)	54 (35.5)
**NO**	271 (72.8)	58 (67.4)	56 (51.4)	15 (65.2)	35 (62.5)	98 (64.5)
**Elevated LDH**						
**YES**	104 (26.3)	12 (14.3)	40 (38.5)	2 (11.1)	16 (29.1)	34 (23.6)
**NO**	292 (73.3)	72 (85.7)	64 (61.5)	16 (88.9)	39 (70.9)	110 (76.4)
**ECOG score**						
**0–1**	438 (95.6)	87 (88.8)	89 (80.2)	20 (83.3)	48 (80.0)	88 (74.6)
**2–4**	20 (4.4)	11 (11.2)	22 (19.8)	4 (16.7)	12 (20.0)	30 (25.4)
**Median WBC**	25.9 (1.2–401.5)	5.4 (0.8–98.7)	14.0 (0.7–283.8)	8.1 (0.9–76.8)	8.9 (0.6–84.1)	12.8 (0.9–180.0)
**Median β2-MG**	3.2 (1.16–22.9)	4.2 (0.2–14.5)	4.4 (1.5–11.9)	3.2 (1.7–6.6)	3.3 (1.5–7.5)	2.98 (1.1–24.5)

Abbreviation: MG: microglobulin, LDH: lactic dehydrogenase, Del deletion.

### The cytogenetic aberrations in each B-CLPD subgroup

The cytogenetic aberrations identified in each B-CLPD subgroup are shown in Table 2. The incidence of del 13q, del11q, trisomy 12 and del17p differed between CLL and non-CLL groups. Del13q is the most affected chromosome in CLL, while del17p was universally detected in each subtype. As SMZL and NMZL are both belong to marginal zone lymphoma (MZL), we grouped them as MZL in the following analysis.

**Table 2:** The cytogenetic aberrations in each B-CLPD subtype

Diseases	Del13q (%)	Trisomy 12 (%)	Del11q (%)	Del 17p (%)
**CLL**	175/447 (39.1)	70/330 (21.2)	46/403 (11.4)	66/454 (14.5)
**B-PLL**	4/6 (66.7)	0/6	0/6	4/6 (66.7)
**SMZL**	9/82 (11.0)	6/56 (10.7)	3/75 (4.0)	4/83 (4.8)
**HCL**	0/23	0/16	0/19	1/23 (4.3)
**WM**	5/93 (5.4)	4/57 (7.0)	2/72 (2.8)	8/90 (8.9)
**NMZL**	3/21 (14.3)	1/10 (10.0)	0/16	4/24 (20.0)
**FL**	5/52 (9.6)	5/35 (14.3)	2/45 (4.4)	1/54 (1.9)
**BCLPD-U**	16/109 (14.7)	8/71 (11.3)	4/96 (4.2)	10/113 (8.8)

Abbreviaton: Del, deletion; t, translocation.

### The prognostic role of the FISH panel was different in each B-CLPD subgroup

The median follow-up was 46.0 months (2–288.0) in CLL. In total, 107 patients died (23.4%). For patients with BCLPD-U, the median follow-up was 36.5 months (range 3.0–96.0), and thirteen of the 115 patients (11.0%) died. The median follow-up for patients with other B-CLPD was 38.0 (range 3.0–239.0) and 49 patients (16.7%) were died.

**Figure 1:**
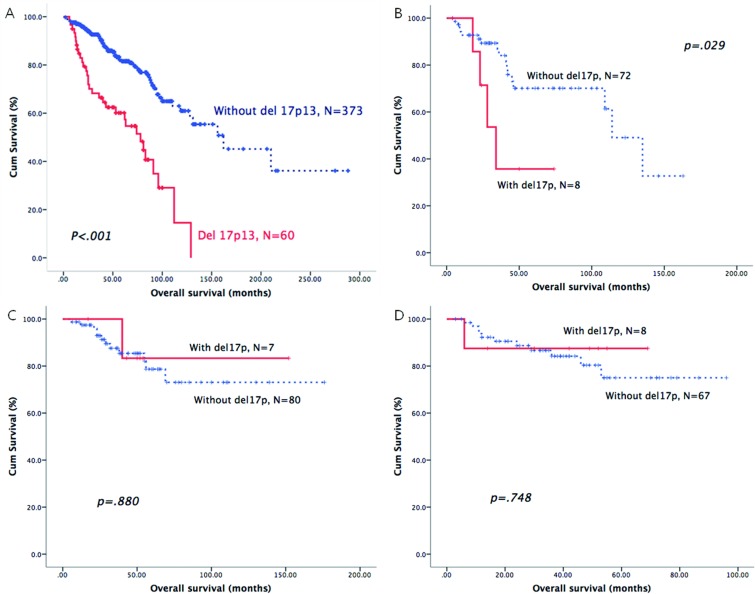
The prognostic role of del17p in patients with different B-CLPD subtypes: CLL (**A**), WM (**B**), MZL (**C**) or BCLPD-U (**D**).

**Figure 2:**
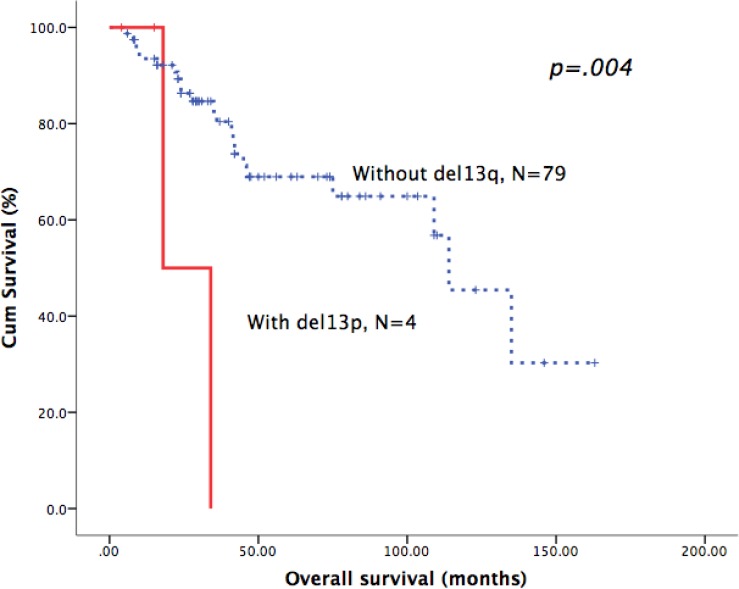
Patients with del13q in WM had poor overall survival

**Figure 3:**
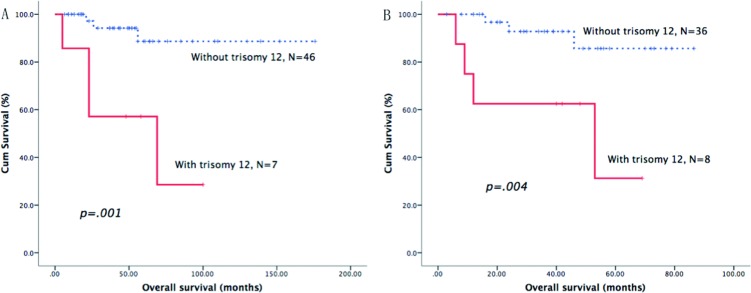
Survival curves of trisomy 12 Patients with trisomy 12 had adverse overall survival in MZL (**A**) and BCLPD-U group (**B**).

As shown in Table 3, we identified the prognostic role of the above-mentioned cytogenetic aberrations in each B-CLPD subgroup. In CLL patients, del17p was the only cytogenetic factor that was an adverse predictor of OS (Figure 1A). In patients with WM, del13q, del11q and del17p were all adverse factors for OS (Figures 1B and 2). In BCLPD-U and MZL patients, trisomy 12 was an adverse prognostic factor for OS (Figure 3). However, del17p did not impact the survival in both MZL and BCLPD-U (Figure 1C, 1D).

**Table 3:** The prognostic role of each cytogenetic aberration in subgroups of B-CLPD

Cytogenetic aberrations	CLL			WM			MZL			BCLPD-U			HCL			FL		
	*N*	Median OS (95% CI)	*P*	*N*	3 years’ OS rate	*P*	*N*	3 years’ OS rate	*P*	*N*	3 years’ OS rate	*P*	*N*	3 years’ OS rate	*P*	*N*	3 years’ OS rate	*P*
**Del 13q**			.161			.004			.385			.670						.258
**YES**	171	129.0 (115.1–143.0)		4	0%		7	83.3 ± 15.2%		13	83.1 ± 11.0%					4	100%	
**NO**	258	129.0 (84.7-173.3)		79	81.8 ± 4.9%		77	90.2 ± 3.9%		61	84.7 ± 5.1%					35	30.2 ± 23.5%	
**Trisomy 12**			.758			.310			.001			.004						.433
**YES**	69	118.5 (66.8–170.2)		4	75.0 ± 21.7%		7	57.1 ± 18.7%		8	62.5 ± 17.1%					3	100%	
**NO**	256	129.0 (not estimated)		46	88.8 ± 5.4%		46	94.2 ± 4.0%		36	92.8 ± 4.9%					23	95.2 ± 4.6%	
**Del 11q**			.098			.041			.589			.092						
**YES**	46	94.0 (56.6–131.4)		2	50.0 ± 35.4%		2	100%		4	66.7 ± 27.2							
**NO**	347	129.0 (96.6-161.4)		61	88.4 ± 4.5%		72	89.6 ± 4.1%		61	86.1 ± 5.0%							
**Del17p > 6.5%**			.000			.029			.880			.748						
**YES**	60	78.0 (55.6–1000.4)		8	35.7 ± 19.8%		7	100%		8	87.5 ± 11.7%							
**NO**	373	162.0 (96.7–227.3)		72	86.7 ± 4.5%		80	87.6 ± 4.2%		67	84.2 ± 4.9%							
**Del17p > 20%**			.000			.087			.998			.286						
**YES**	46	62.0 (32.6–91.4)		2	50.0 ± 35.4%		6	100%		5	100%							
**NO**	381	162.0 (96.9–227.1)		78	79.8 ± 5.4%		79	87.5 ± 4.2%		70	83.3 ± 4.9%							

Abbreviation: Del: deletion, T: translocation, MZL includes SMZL and NMZL with bone marrow involvement.

As the cut-off 20% of del17p is regarded as more power to predict the adverse survival in CLL, we used this cut-off value to recalculate the survival impact of del17p in these B-CLPD subgroups. CLL patients with del17p >20% had a shorter median OS. However, del17p >20% still had no significant impact on the survival of patients with other B-CLPD subtypes (Table 3).

The survival and clinical / cytogenetic characteristics of B-PLL were shown in Supplementary Table 1. The overall survival of B-PLL was poor, with an estimated OS 12.0 months (95% CI 9.9–14.1), but no conclusion could be made about the prognostic role of cytogenetic aberrations in B-PLL due to little cases. The FL or HCL patient with del17p was also described in Supplementary Table 1.

### The multivariate analysis of the prognostic factors in different B-CLPD subtypes

We then analyzed other factors that might predict the survival of B-CLPD patients in the each subtype group. As shown in Table 4, B symptoms, elevated LDH and high Rai risk all adversely affected the OS of CLL patients. For patients with other B-CLPD, male, older than 60 years and elevated LDH were the adverse factors for OS. In patients with BCLPD-U, only elevated LDH was a poor prognostic factor for OS.

**Table 4:** The univariate analysis of the prognostic significance of clinical characteristics in subgroups of B-CLPD

Clinical characteristics	CLL			WM			MZL			BCLPD-U		
	*N*	Median OS (95% CI)	*P*	*N*	3 years’ OS rate	*P*	*N*	3 years’ OS rate	*P*	*N*	3 years’ OS rate	*P*
**Sex**			.995			.380			.264			.174
**Male**	296	129.0 (88.6–169.4)		65	78.0 ± 5.8%		43	84.0 ± 6.0%		52	79.5 ± 6.2%	
**Female**	141	128.0 (100.6–155.4)		23	78.9 ± 11.4%		47	94.0 ± 4.1%		25	92.0 ± 5.4%	
**Age > 60 years**			.408			.290			.318			.965
**YES**	185	Not reached		46	78.1 ± 7.0%		29	86.5 ± 7.3%		35	84.9 ± 6.2%	
**NO**	253	129.0 (108.8–149.2)		42	78.9 ± 7.4%		61	90.4 ± 4.1%		42	81.8 ± 6.9%	
**B symptom**			.001			.277			.356			.598
**YES**	96	90.0 (76.3–103.7)		25	75.2 ± 9.7%		42	86.0 ± 5.9%		22	77.3 ± 8.9%	
**NO**	262	129.0 (94.1–163.9)		53	76.6 ± 6.7%		47	91.4 ± 4.8%		49	84.8 ± 5.8%	
**Elevated LDH**			.000			.009			.002			.078
**YES**	93	89.0 (70.1–107.9)		11	42.1% ± 17.7%		32	74.7 ± 9.2%		15	64.4 ± 12.9%	
**NO**	284	162.0 (102.8–221.2)		64	83.3 ± 5.7%		55	95.8 ± 2.9%		60	88.6 ± 4.4%	
**Rai risk stage**												
**Low**	66	Not reached	.000									
**Intermediate**	230	128.0 (86.7–169.3)										
**High**	141	97.0 (73.7–120.3)										
**Splenomegaly**			.192			.865			.628			.326
**YES**	165	100.0 (66.0–134.0)		30	73.1 ± 10.6%		76	86.5 ± 4.5%		37	84.0 ± 6.6%	
**NO**	211	131.0 (not estimated)		57	80.4 ± 5.7%		13	100%		36	81.1 ± 7.2%	
**Hepatomegaly**			.056			.956			.899			.911
**YES**	25	97.0 (63.2–130.8)		14	85.7 ± 9.4%		14	90.0 ± 9.5%		6	83.3 ± 15.2%	
**NO**	367	129.0 (72.3–185.7)		71	75.4 ± 6.6%		75	88.7 ± 4.1%		67	82.6 ± 5.1%	
**ECOG score**			.112			.330			.082			.278
**0–1**	368	128.0 (114.2–141.8)		78	76.3 ± 5.5%		72	90.4 ± 4.2%		57	87.2 ± 4.5%	
**2–4**	16	54.0 (51.5–56.5)		9	100%		16	81.3 ± 9.8%		19	63.1 ± 16.7%	

A Cox model was then constructed for a multivariate analysis of the prognostic factors in patients with CLL, other B-CLPDs or BCLPD-U using the significant predictors identified in the univariate analysis mentioned above. As shown in Table 5, elevated LDH, high Rai risk and del17p were independent predictors of OS in CLL. For patients with other B-CLPDs, elevated LDH, age >60 years and del13q were independent adverse predictors of OS. In BCLPD-U, trisomy 12 was the only independent factor for adverse OS, with a 10-fold relative risk.

**Table 5:** Multivariate analysis of prognostic factors for overall survival in CLL and other B-CLPD groups

Disease groups	Number of patients	Factors	*P*	RR	95% CI
CLL	*N* = 296	B symptom	.138	1.4	0.9–2.2
		Elevated LDH	.008	1.8	1.2–2.9
		Rai risk stage	.012	1.6	1.1–2.3
		De 17p	.000	2.4	1.5–-3.9
WM^*^	*N* = 65	Del13q	.515	2.0	.2–16.7
		Del17p	.256	2.6	.5–14.2
		Elevated LDH	.046	3.1	1.0–9.3
MZL	*N* = 51	Elevated LDH	.950	1.0	.2–4.7
		Trisomy 12	.005	10.0	2.0–49.7
BCLPD-U	*N* = 42	Elevated LDH	.848	1.2	0.2–6.6
		Trisomy 12	.018	6.3	1.3–29.2

Abbreviation: LDH: lactic dehydrogenas, Del deletion, CI: confidence interval.

^*^del11q was not included because only two patients with del11q in WM.

### Comparison of the survival of patients with del17p between B-CLPD subgroups

**Figure 4:**
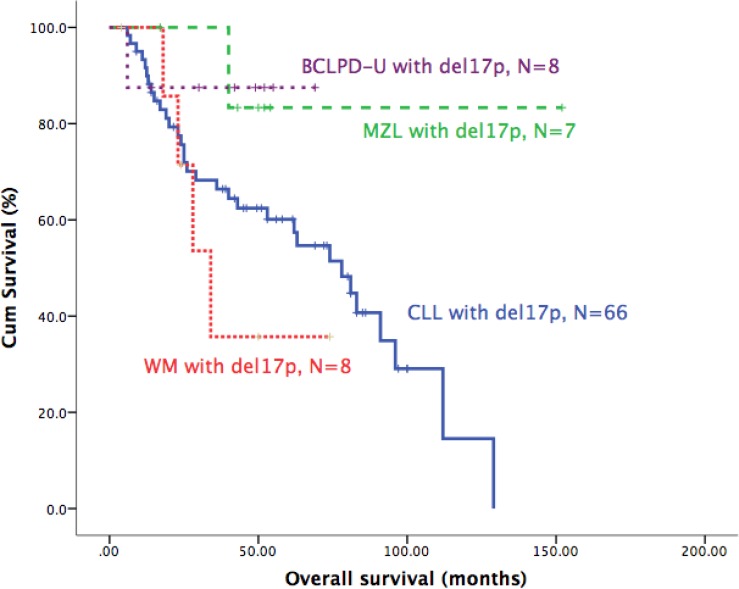
Survival comparisons of patients with del17p in different B-CLPD subtypes

As del17p had different prognostic role in different B-CLPD subgroups, we directly compared the clinical characteristics and survival between CLL and other B-CLPDs. As shown in Table 6, there was no significant difference in aspect of clinical characteristics. The survival of MZL and BCLPD-U was better than CLL and WM (Figure 4).

**Table 6:** Comparison of clinical characteristics between B-CLPD subgroups with del17p

Clinical characteristics	CLL *N* = 66 (%)	WM *N* = 8 (%)	MZL *N* = 8 (%)	BCLPD-U *N* = 10 (%)	*P*
**Median age (range)**	54.5 (39.0–86.0)	63.5 (40.0–74.0)	55.5 (41.0–79.0)	62.5 (49.0–73.0)	.291
**Age > 60 years**					.205
**YES**	21 (31.8)	5 (62.5)	3 (37.5)	6 (60.0)	
**NO**	45 (68.2)	3 (37.5)	5 (62.5)	4 (40.0)	
**Sex**					.107
**Male**	44 (66.7)	7 (87.5)	5 (62.5)	10 (100.0)	
**Female**	22 (33.3)	1 (12.5)	3 (37.5)	0	
**B symptom**					.870
**YES**	24 (40.0)	2 (28.6)	4 (50.0)	4 (40.0)	
**NO**	36 (60.0)	5 (71.4)	4 (50.0)	6 (60.0)	
**Elevated LDH**					.610
**YES**	25 (41.7)	1 (16.7)	3 (57.1)	3 (30.0)	
**NO**	35 (58.3)	5 (83.3)	4 (42.9)	7 (70.0)	
**Median WBC (×10**^9^**/L)**	36.9 (2.8–359.3)	5.7 (3.0–98.7)	19.8 (9.2–79.8)	24.7 (4.2–168.2)	.083
**Median** **β2-MG(mg/L)**	3.7 (1.52–22.9)	5.3 (2.5–6.4)	4.5 (2.4–8.4)	2.6 (1.9–9.5)	.538

Abbreviation: LDH: lactic dehydrogenas, Del deletion, MG: macroglobulin, WBC: white blood cell.

## DISCUSSION

As in other types of cancer, cytogenetic aberrations play an important role in the lymphoma pathogenesis of each subtype. For example, the hallmark cytogenetic aberration in FL is t(14;18) (q32;q21); the deletion of 7q32 was reported in 40% of SMZL patients [6, 7]; The best-known chromosomal change in WM is the deletion of 6q [8]. However, the prognostic role of cytogenetic aberrations has not been systematically investigated in B-CLPD, except CLL. For example, deletions of 1p, 6q, and 17p and gains of 7 and 12q have been strongly associated with a poor prognosis in FL [9–11], but certain studies have failed to show a link between these secondary cytogenetic changes and outcome [12]. WM patients with 6q deletions have higher levels of β2-microglobulin and monoclonal paraprotein and a greater tendency to display anemia and hypoalbuminemia, but no correlation between the deletion of 6q and survival has been established [13, 14]. The most frequent incidence of del 7q and +3/3q+ in SMZL did not impact the survival in a large analysis [7, 15]. In addition, the prognostic impact of cytogenetic abnormalities has been infrequently assessed in NMZL, B-PLL and HCL patients. Moreover, the techniques that were used to detect the cytogenetic aberrations mentioned above are usually conventional karyotyping and array comparative genomic hybridization (aCGH). The low sensitivity of karyotyping and the poor accessibility of aCGH have limited their use in routine clinical practice. FISH, a useful tool for the detection of cytogenetic aberrations in cancer, has been used less frequently in these B-CLPD subtypes because there is no readily available DNA probes (commercial probe) for such rare abnormal chromosome site.

Detection of cytogenetic aberrations by FISH has been the critical practice in CLL to determine the treatment choice and prognosis [5]. This FISH panel includes del13q, del11q, del17p, trisomy 12 and IGH translocation. CLL patients with del(17p) and del(11q) always have the shortest median survival, while the deletion of 13q as the sole abnormality predicts a favorable outcome in CLL [3, 16]. However, the prognostic role of this FISH panel has never been systematically evaluated in other B-CLPDs, which always have similar clinical feature with CLL. In this study, we first investigated the prognostic efficacy of this CLL FISH panel in other B-CLPD subtypes with a large series patient and unexpected results were founded.

Del17p is now the highest risk factor for CLL and it makes the treatment of such patients challenging. Del17p is also an independent adverse factor in MCL [15, 17], an aggressive subtype of B-CLPD, and an important adverse prognostic factor in many other hematological malignances, such as multiple myeloma [18]. However, surprisingly, del17p was not an adverse predictor in some B-CLPD subtypes in the current study, such as MZL and unclassified B-CLPD. The cut-off value of del17p at 20% was identified to be more powerful to predict the adverse survival of CLL [19]. We use the cut-off value at 20% to evaluate the prognostic role of del17p in other B-CLPD subtypes. However, deletion of 17p more than 20% still had no significant impact on the survival of non-CLL B-CLPD groups. The incidence of 17p (TP53) deletions in WM was 8% and indicated a short progression-free survival and short disease-free survival in a series of 174 WM patients [14], which accorded with our results. The deletion of 17p in MZL also had been reported with no impact on survival of MZL in another large series study (218 patients) [20]. In another multicenter study, TP53 deletion acted as an adverse factor of survival in univariate analysis but lost in the multivariate analysis in SMZL patients [7].

The adverse prognosis associated with del17p is related to its effects on the p53 pathway. It would therefore be of interest to examine whether patients with a minor poor clone exhibited alterations in the p53 pathway (i.e., mutations in ATM or the p53 allele). However, the absence of suitable patient specimens precluded these experiments in the present retrospective study. In clinical practice, CLL patients with del17p are considered as ultra-high risk group and are treated using an extraordinary strategy, such as allogeneic stem cell transplantation [21]. As the treatment of some other indolent B-CLPD always imitates CLL treatment [22], this therapeutic strategy for CLL patients with del17p should carefully apply to other B-CLPD.

Deletion of 13q14 is the most frequent genomic aberration in CLL and it is also a common additional cytogenetic aberration in approximately 10%–43% of patients with MCL [23, 24]. In addition, 9%–16% of patients with WM [25, 26] and 12 out of 239 (5%) SMZL patients [7] carry the 13q14 deletion, as determined by FISH. In the present study, del13q was a common cytogenetic aberration in each subtype of B-CLPD (Table 2), except for HCL. This is the first study to systematically describe the distribution of del 13q in B-CLPD, and especially sole del13q, is an advantageous factor for survival in CLL. However, in MCL, del13q is an adverse prognostic factor [24, 27]. And in some studies of WM with few patients (22 to 37 patients), del13q had no survival impact [25, 26]. In the current study, we declared del13q to be a prognostic factor for poor survival in patients with WM, but finally lost the prognostic role in the multivariate analysis. This interesting phenomenon may reflect the different roles of del13q in the pathogenesis of different B-CLPD subtypes. There are several putative genes in the region of 13q14 [28] and deletions of different sizes in this region may involve different genes. Thus, detailed work should be conducted to distinguish del13q in each B-CLPD.

Although MZL always shows trisomy 12,3 and 18 in many studies, no study had proved the trisomy 12 adversely impacted the survival of MZL [7, 29]. In this analysis, we found that trisomy 12 was an independent poor prognostic factor for MZL patients. In CLL, trisomy 12 is associated with atypical morphologic and/or immunophenotypic features [30] and tends to Richter transform [31]. The clinical association of trisomy 12 with other B-CLPD has not been established, but trisomy 12 does not impact the survival of FL [32] or SMZL [7] patients, in whom a high frequency of trisomy 12 was observed in the present study (Table 2).

The differential diagnosis of B-CLPD is a challenge in routine clinical practice. Although most patients can be diagnosed using diverse methods, such as flow cytometric immunophenotyping, cytogenetic examination and biopsy [2, 33], in a minority of patients, no specific pathological diagnosis can be made [2, 34, 35]. In this study, we defined these patients as BCLPD-U. All of these patients with BCLPD-U had an atypical immunophenotype as determined by flow cytometry, were negative for t(11;14)(q13;q34) by FISH, and had undergone a bone marrow biopsy. Atypical CLL was specifically excluded by combined immunophenotypical and morphological feature in this group, based on the criteria previously mentioned [36, 37]. These patients may have fit into one of the previously well-defined WHO categories if more biopsy characteristics could have been identified [38], but an enlarged lymph node biopsy was always not available for these patients. Thus, the entity was heterogeneous. Because BCLPD-U is a new putatively classified entity that was not previously described, we investigated it separately here. The clinical characteristics and cytogenetic aberrations were similar between the BCLPD-U group and other B-CLPD groups (Table 1). One prior study analyzed the prognostic factor for unclassified B-CLPD and found that an elevated serum LDH level and an age of >60 years retained their prognostic relevance for OS in 156 patients [35]. In this study, we also identified elevated LDH as an independent prognostic factor for the BCLPD-U. However, age did not influence the survival in this study. Although the frequencies of cytogenetic aberrations in the panel were similar between other B-CLPD groups and BCLPD-U group, the clinical impact was different in this study. Del13q did not impair the survival of BCLPD-U patients; in contrast, trisomy 12 predicted poor overall survival, with a 6-fold relative risk.

Finally, we noticed that the constituent of this cohort is not according with the incidence of each B-CLPD subtype. For example, the MALT lymphoma and FL should take the major of B-CLPDs, but we did not include MALT lymphoma here and FL only occupied 7%. Two main reasons should be considered to explain this bias. First, the incidence of B-CLPDs is different between the Western and the Eastern population. For example, FL is less common in the East. Second, Our hospital is a specialized hospital focusing on hematology and patients with bone marrow or peripheral blood involvement were preferentially enrolled in, while patients with solid tumor as the major presentation always chose Cancer Hospital. And patients with MALT lymphomas often originally diagnosed or treated at other departments, such as gastroenterology, ophthalmology department, but not hematology department. However, we included all of the available B-CLPD patients in our hospital and no selection in this analysis.

In conclusions, we first systematically investigated the cytogenetic aberrations in a large series of unselected B-CLPD patients using the CLL FISH panel. We found that del17p had no impact on the survival of patients with MZL and BCLPD-U, although it impaired the survival of CLL patients. Instead, del13q was a poor predictor of the survival of patients with WM and trisomy 12 was an independent adverse factor in these BCLPD-U patients. This study indicates that different cytogenetic aberrations should be used to predict survival in different subtypes of B-CLPD and the most important point is that the treatment strategy used for CLL patients with del17p should be carefully applied to other B-CLPD with del17p.

## METHODS

### Patients

The study cohort consisted of 875 untreated B-CLPD patients from the Institute of Hematology and Blood Disease Hospital, Chinese Academy of Medical Sciences, and the Peking Union Medical College (CAMS & PUMC) with retrospective analysis. A diagnosis of CLL or other B-CLPD subtypes was made according to the World Health Organization (WHO) classification [1, 39]. Flow cytometric analysis, bone marrow biopsy, immunohistochemistry (IHC), t(11;14)(q31;q34) analysis by FISH, and lymph node biopsy (if available) were carried out as routine clinical practice for diagnosis. Some of the patients could not be classified into any of the established categories based on the bone marrow biopsy, morphology, flow cytometric analysis, molecular and/or cytogenetic exams, and lack of available histological specimen, such as lymph node. We classified these patients as BCLPD-U.

All of the patients provided written informed consent in accordance with the requirements of the Declaration of Helsinki. This study was approved by the Ethics Committee of Blood Disease Hospital, Chinese Academy of Medical Sciences (NI2016001-EC-1). The treatment during the course of the disease includes chlorambucil, fludarabine ± cyclophosphamide, CHOP and COP, combined with or without rituximab. The indications for treatment were standardized per the iwCLL criteria [40], GELF criteria for FL [41] and IWWM criteria for WM [42].

### Fluorescence *in situ* hybridization

An interphase FISH analysis was performed on peripheral blood or bone marrow samples at diagnosis. The CLL FISH “panel” included probes for the loci centromere 12 (CEP12), 13q14.3 (LSI D13S25/*RB-1*), 14q32 (LSI IGHC/IGHV), 17p13 (LSI *TP53*), and 11q22 (LSI *ATM*). Sample preparations and hybridizations were conducted following the manufacturer’s recommendations and as previously described [43]. An LSI *CCND1/IGH* Dual Color, Dual Fusion Translocation Probe was used to exclude the possibility of mantle cell lymphoma (MCL) in the case with t(14q32) positivity. All of the probes were purchased from Vysis (Abbott Co, Downers Grove, IL, USA). Signal screening was carried out on at least 200 cells with well-delineated signals. The cut-offs for positive values (the mean of the normal control + 3 SDs) were determined using samples from ten cytogenetically normal people and were 7.5% for CEP 12 (trisomy 12); 6.5% for deletions of 13q13 (D13S25 and *RB1*), 11q22 (*ATM*) and 17p13 (*TP53*); and 4.5% for t(14q34) (IGH translocation) and t(11;14)(q13;q34) (*CCND1/IGH*).

### Survival and statistical analysis

Overall survival (OS) was measured as the interval between the date of diagnosis or presentation of obvious disease-related symptoms and the date of death or last follow-up. Fisher’s exact test or the chi-square test was used to determine the statistically significant differences between the clinical characteristics of the two groups. Survival curves were constructed using the Kaplan-Meier method, and prognostic features were evaluated by univariate analysis (i.e., the log-rank test). The effects of potential prognostic variables on survival were assessed according to the Cox regression method for multivariate analysis. *P* values < 0.05 were considered statistically significant. All of the calculations were performed using the SPSS statistical software package (version 13.0, SPSS Inc., Chicago, IL. USA).

## SUPPLEMENTARY MATERIALS TABLE


