# Supplementary material for: Del17p does not always significantly influence the survival of B-cell chronic lymphoproliferative disorders
Source: Oncotarget. 2017 Dec 15;9(3):3353–64. doi: 10.18632/oncotarget.23261 (PMC5790468; doi:10.18632/oncotarget.23261)
Supplement: Supplementary file 1 [file oncotarget-09-3353-s001.pdf]

# Del17p does not always significantly influence the survival of B-cell chronic lymphoproliferative disorders

## SUPPLEMENTARY MATERIALS

**Supplementary Table 1: The clinical and cytogenetic characteristics and survival of patients with B-PLL and FL/HCL patients with del17p**

| No. | Sex | Age | Del 13q | Del 17p | Survival time (months) | status |
|---|---|---|---|---|---|---|
| **B-PLL1** | male | 60 | pos | pos | 11 | death |
| **B-PLL2** | male | 68 | pos | neg | 14 | Alive |
| **B-PLL3** | male | 74 | pos | pos | 12 | death |
| **B-PLL4** | female | 50 | neg | neg | 6 | death |
| **B-PLL5** | male | 83 | pos | pos | 24 | death |
| **FL** | Male | 30 | pos | pos | 56 | Alive |
| **HCL** | Male | 49 | neg | pos | 3 | death |

Abbreration: pos, positive; neg, negative.
